# A Cryptic Subterranean Mammal Species, the Lesser Blind Mole Rat (*Nannospalax leucodon syrmiensis*)—Retreated but Not Extinct

**DOI:** 10.3390/ani14050774

**Published:** 2024-02-29

**Authors:** Vanja Bugarski-Stanojević, Marko Đokić, Gorana Stamenković, Nataša Barišić Klisarić, Oliver Stojković, Vida Jojić, Ivo Savić

**Affiliations:** 1 Department of Genetic Research, Institute for Biological Research “Siniša Stanković”—National Institute of Republic of Serbia, University of Belgrade, 11108 Belgrade, Serbia; 2 Department of Evolutionary Biology, Institute for Biological Research “Siniša Stanković”—National Institute of Republic of Serbia, University of Belgrade, 11108 Belgrade, Serbia; 3 Institute of Forensic Medicine, Faculty of Medicine, University of Belgrade, 11060 Belgrade, Serbia; 4 Institute of Zoology, Faculty of Biology, University of Belgrade, 11060 Belgrade, Serbia

**Keywords:** endangered species, genetic diversity, habitat loss, habitat fragmentation, rodents, *MT-CYTB*, *16SrRNA*, chromosomal speciation, conservation, biodiversity

## Abstract

**Simple Summary:**

Due to adaptations to a completely subterranean lifestyle, blind mole rats have become an interesting research model for longevity and cancer resistance. The lesser blind mole rat from the genus *Nannospalax* is additionally characterized by extensive chromosomal changes, with 74 chromosomal forms described. As a result of these changes and of their morphological similarity, their taxonomy is unclear; consequently, many unrecognized species are endangered due to habitat fragmentation and reduction. Their official conservation status is still undefined due to insufficient data. Among the 25 chromosomal forms of *N. leucodon* in Europe, five have been identified as completely reproductively isolated and genetically divergent cryptic species in Serbia. The most endangered, *N. l. syrmiensis*, which was described 50 years ago as a group of populations endemic to Serbia, has been declared extinct in the literature. Using nucleotide comparison of two mitochondrial gene segments between old, archived and recently sampled material, we provide evidence that *N. l. syrmiensis* is not extinct. However, it has disappeared in a large part of its former range, mainly due to urbanization, invasive agriculture, and treatment as a pest. In order to preserve biodiversity, detailed monitoring, population-structure studies, risk assessment and appropriate conservation measures are required.

**Abstract:**

Blind mole rats (genus *Nannospalax*) attract a great deal of attention because of their cancer resistance and longevity. Due to the high rate of chromosome rearrangements, 74 *Nannospalax* chromosomal forms have been discovered. The convergence of their external morphology complicates their taxonomy, and many cryptic species remain unrecognized. Thus, the European *N. leucodon* supersp. is listed in the IUCN Red List of Threatened Species with “Data Deficient” status. It is crucial for the conservation of biodiversity to clarify its taxonomy, to recognize each cryptic species, and assign to them the correct conservation status. Of the more than 20 chromosomal forms described within *N. leucodon*, five cryptic species occur in Serbia. The most threatened among them—*N. l. syrmiensis*, described and named 50 years ago in the regions of Srem, Belgrade and Mačva—has been declared extinct in the literature, which may have negative consequences for the conservation of wildlife genetic diversity. Through five years of fieldwork and comparison of *16SrRNA* and *MT-CYTB* gene segments between old, archived teeth and recently collected material, we show that *N. l. syrmiensis* is not extinct. However, its habitat has been fragmented and reduced, owing primarily to anthropogenic impact. Therefore, detailed surveillance, population-structure studies, risk assessment, and appropriate conservation measures are needed.

## 1. Introduction

A clade of blind mole rats (BMRs) Spalacidae, Rodentia, draws constant attention in several scientific fields due to their numerous adaptations to a subterranean environment. They are small, solitary, exclusively subterranean, and highly specialized mammals with an extreme tolerance to hypoxia and hypercapnia, specific blood characteristics, high vessel density, and other physiological and morphological modifications. With a lifespan of about 20 years and a unique resistance to cancer [1,2,3,4,5], BMRs are the only long-lived and cancer-proof rodents in Europe.

**Figure 1:**
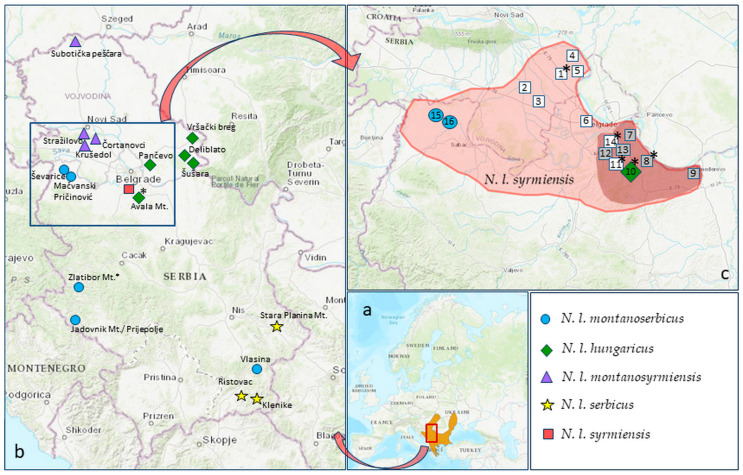
Geographical distribution of five *N. leucodon* cryptic species from Serbia. (**a**) Distribution area of *N. leucodon* supersp. (**b**) Capturing localities in Serbia, including our previously published data (listed in Table S1): *N. l. syrmiensis*—square; *N. l. montanosyrmiensis*—triangle; *N. l. hungaricus*—rhomb; *N. l. montanoserbicus*—circle; *N. l. serbicus*—star. (**c**) Distributional area of *N. l. syrmiensis* according to [11,19]—light red; distribution according to our research—dark red. Localities: 1—Stara Pazova, 2—Donji Petrovac, 3—Krnješevci, 4—Slankamen, 5—Stari Banovci, 6—Zemun Polje, 7—Višnjica, 8—Vinča, 9—Udovice (Smederevo), 10—Avala, 11—Jajinci, 12—Banjica, 13—Košutnjak, 14—Banovo Brdo, 15—Mačvanski Pričinović (Bogatić), 16—Ševarice (Bogatić); empty squares—BMR not present; filled squares—fresh samples; *—old, archived samples. Source of the map: IUCN (International Union for Conservation of Nature) 2011. *Nannospalax leucodon*. The IUCN Red List of Threatened Species. Version 2023-1. Accessed on 8 February 2024.

These vastly unusual rodents are represented by two genera: the greater BMR, *Spalax* (Guldenstaedt 1770), and the lesser BMR, *Nannospalax* (Palmer 1903). The latter represents an excellent research model for chromosomal speciation studies, as the genus *Nannospalax* includes 74 groups with fixed chromosomal differences [6,7,8] with a wide range of diploid chromosomal number (2*n*), from 36 to 60. These chromosomal forms (CFs) are alternatively labelled as cytotypes, races, or sibling/good biological/cryptic species [8,9,10]. Another complicating circumstance in species delimitation in this genus is the species’ convergent morphology—reduced interspecific phenotypic variability that most likely developed as a result of stabilizing selection modeled by extreme environmental settings [11]. Biometric and/or morphological studies of skulls, jaws and teeth are a valuable source for distinguishing living mammal species in general, while morphological data, especially fossil teeth, can provide valuable insights into the evolutionary history and paleobiology of mammals. However, they are not reliable for species identification in morphologically convergent taxa such as the cryptic species of the lesser BMR. Therefore, the number of recognized species in the genus *Nannospalax* ranges from only one [12] to 14 [11]. The International Union for Conservation of Nature (IUCN) Red List of Threatened Species recognizes three morphospecies/superspecies, out of which the European species is classified as Data Deficient (DD) [13]: (1) the lesser BMR *N. leucodon* (Nordmann 1840) from parts of central and South-Eastern Europe (Figure 1a); the other two, classified as Least Concern (LC), are (2) the Anatolian BMR *N. xanthodon* (Nordmann 1845) (synonym: *N. nehringi* (Satunin 1898)), which occurs in Transcaucasia, most of Turkish Anatolia and certain East Aegean islands; and (3) the Palestine BMR *N. ehrenbergi* (Nehring 1898), which occurs in southeastern Anatolia in Turkey, Iraq, Syria, Lebanon, Israel, Jordan, and Egypt. Each of these three superspecies comprises more than 20 CFs, with several reproductively isolated and genetically well-differentiated cryptic species [8,14,15,16]. Some of them are already seriously endangered due to the loss and fragmentation of their natural habitats [6,10,17]. In most countries, there is no targeted species protection plan because of their uncertain conservation status, which results from insufficiently clarified taxonomy and cryptic speciation.

The lesser blind mole rat, *Nannospalax leucodon* (Nordmann 1840) superspecies, is endemic to Europe, with 25 CFs reported so far [6,8]. They are known as typical inhabitants of the steppe-like grasslands, mountain steppes, and sand steppes, and they avoid marshy areas and quicksand [18]. Breeding experiments that revealed the complete reproductive isolation of seven *N. leucodon* CFs [8,11,19], substantiated by the results of molecular phylogenetic investigations [14,15,16,20,21], strongly suggest their cryptic-species status. The extent of their evolutionary divergence resembles that recorded among clearly distinguishable *Spalax* species [14,21].

All five cryptic species of *N. leucodon* distributed in Serbia (Figure 1b), *N. l. hungaricus* (Nehring 1898); *N. l. serbicus* (Méhely 1909); *N. l. montanoserbicus* (Savić and Soldatović 1974); *N. l. syrmiensis* (Méhely 1909) and *N. l. montanosyrmiensis* (Savić and Soldatović 1974), which were documented 50–60 years ago [8], are reproductively completely isolated and meet the criteria to be classified as distinct species. Although *N. leucodon* has strictly protected status in Serbia, as proposed by Vasić et al. [22], the natural habitats of Serbian *N. leucodon* cryptic species have been increasingly reduced, mostly due to anthropogenic influences, i.e., extensive urbanization and invasive agriculture. Despite their national protected status, they are also treated as pests and killed. In a previous publication [21], we drew attention to two cryptic species with highly reduced and fragmented ranges, *N. l. syrmiensis* and *N. l. montanosyrmiensis*, categorized them as Potentially Endangered/Critically Endangered, and emphasized the need for detailed monitoring and population surveys.

Here, we report on our study of *N. l. syrmiensis*. During extensive cytogenetic mapping of BMRs in the 1970s, Soldatović [19,23] and Savić and Soldatović [24] described the BMR from the Srem district as a distinct CF, i.e., a group of seven populations with the diploid chromosome number 2*n* = 54 and the number of chromosomal arms *NF* = 90, and named it *Spalax leucodon syrmiensis* (Méhely 1909). Later, they accepted nomenclature with two genera and proposed a separate species with the name *Nannospalax syrmiensis* (Méhely 1909) on the basis of comparative morphometric, craniometric and karyotypic studies, evidence of reproductive isolation, and the character of surrounding ecogeographic formations [11]. It was widespread in the districts of Srem (*locus typicus*—Stara Pazova) and Mačva, as well as in the Belgrade region [11] (Figure 1c). After the investigations summarized in Savić and Soldatović [11], there have been no published data regarding distribution of *N. l. syrmiensis* since 1988 [25]. However, *N. l. syrmiensis* was declared probably extinct [26], and this statement was repeated [10,15]. Almost 35 years after the last data were published, Bugarski-Stanojević et al. [21] reported newly captured *N. l. syrmiensis* specimens in Serbia. Despite these results, in the latest comprehensive study, which included 22 *N. leucodon* CFs, Németh et al. [16] referred to *N. l. syrmiensis* as a missing taxon and “*incertae sedis*”.

For the conservation of biodiversity, including the genetic diversity of wildlife, declaring a particular cryptic species as probably extinct may have negative consequences. If *N. l. syrmiensis* is absent from the literature, or from part of its range, this does not mean that it is extinct. In this study, we conducted systematic fieldwork over several years/seasons, covering the entire known range of *N. l. syrmiensis*. For species confirmation, we compared the nucleotide sequences of two mitochondrial gene segments (*16SrRNA* and *MT-CYTB*) between newly captured animals and old archived tooth samples previously identified as *N. l. syrmiensis* by karyotyping. The tooth samples (collection Ivo Savić; Institute for Biological Research “Siniša Stanković”—National Institute of Republic of Serbia, University of Belgrade—IBISS) came from the *locus typicus*—Stara Pazova and several other localities in the Belgrade region. Another four cryptic *N. leucodon* species distributed in Serbia are newly sampled and included in this study together with tooth samples to avoid misinterpretation of species identity and account for the possible presence of hybrids. MtDNA genes were selected instead of nuclear genes to increase the success rate of sequencing from old archived material. The mentioned methods have already proven to be excellent for the identification of BMR cryptic species [14,15,16,21]. The *16SrRNA* gene is regularly used to identify well-differentiated species, genera and distantly related taxa [27,28,29]. Polymorphism of the *MT-CYTB* gene has been widely used to identify species and phylogenetic relationships in BMRs [30,31,32]. In addition to proving that *N. l. syrmiensis* still occurs in Serbia, we determined its greatly reduced distribution range.

## 2. Materials and Methods

Field work—Localities previously reported for the occurrence of *N. l. syrmiensis* [11], as well as a wide surrounding area, were surveyed from 2019–2023. During the same period, another four cryptic *N. leucodon* species distributed in Serbia were sampled in eight localities, so that a total of 18 animals were newly collected and sampled from their natural habitat for this study (Figure 1b, Table S1). Our fieldwork included the following: (1) detection of active burrows, which involved noting their distribution/shape; a detailed inspection to distinguish BMRs from moles, e.g., examining the structure of underground tunnel walls and measuring of their diameters, which should be about 5–7 cm [33]; and searching for imprinted animal tracks in the soil and the presence of food stores; (2) capture of live animals by opening their tunnel systems; (3) collection of specimens accidentally killed during capture or by local residents during agricultural activities; and (4) conduction of interviews of citizens. All animal-sampling protocols complied with the current laws of the Republic of Serbia. The Ministry of Environmental Protection of the Republic of Serbia issued annual permits (numbers listed in Institutional Review Board Statement) that allowed the capture and sampling of a limited number of animals per location.

Data collection—After capture, the live animals were transported in separate cages to IBISS and treated by veterinarian with isoflurane inhalation anaesthesia. A fingertip from a hind foot was removed and stored at −80 °C for DNA extraction. After a few days of recovery, each animal was released back into its own underground tunnel system. Liver and muscle tissue from dead animals was stored in absolute ethanol or frozen at −20 °C. Live animals were treated in accordance with Directive 2010/63/EU of the European Parliament and of the Council of 22 September 2010 on the protection of animals used for scientific purposes. All animal procedures were approved by the Ethical Committee for the Use of Laboratory Animals of the Institute for Biological Research “Siniša Stanković”, University of Belgrade (IBISS) and the Veterinary Directorate of the Ministry of Agriculture, Forestry, and Water Economy of the Republic of Serbia (Licence No. 323-07-11307).

The teeth (molars) were extracted from four old, archived skull specimens (Collection Ivo Savić, IBISS). They were karyotyped between 1963 and 1977 [11]. Three were identified as *N. l. syrmiensis*; one of these animals was captured in the Srem district (*locus typicus*—Stara Pazova) in 1969, while the other two were from the Belgrade region—Vinča and Jajinci—and were captured in 1963 and 1977, respectively (Figure 1c, Table S1). A specimen caught in sympatry with *N. l. syrmiensis* from the locality Avala Mt. (1969) was identified as *N. l. hungaricus*.

Total DNA was extracted from finger parts of living animals or from liver and muscle tissue using the DNeasy Blood and Tissue Kit, Qiagen. DNA isolation from teeth was carried out at the Institute of Forensic Medicine according to the procedures described in [14]. Universal primers [34] and seminested PCR were used for the amplification of ~600 bp long 16SrRNA gene fragments, as described in [14]. To obtain longer sequences of DNA extracted from old teeth, *MT-CYTB* gene parts were amplified with several primers that had been newly designed and modified according to [35,36]. Primer sequences and the reaction conditions are those given in [21]. Sequencing was performed in both directions by a third party.

Sequence analysis—Our analysis generated 22 new *16SrRNA* and 20 *MT-CYTB* sequences from five cryptic *N. leucodon* species. They were all visually examined using the FinchTV 1.4.0 chromatogram viewer (Geospiza Inc. Seattle WA, USA), analysed using BioEdit Ver. 7.2.5 [37], and checked for the presence of stop codons/chimeric sequences. Additional sets of 40 *16SrRNA* and 42 *MT-CYTB* sequences were compared using the Basic Local Alignment Search Tool (BLAST) and obtained from GenBank (Table S1). The final *16SrRNA* dataset of 62 individual sequences included a total of 46 individuals from five cryptic *N. leucodon* species, five *N. xanthodon*, eight *N. ehrenbergi*, and three *Spalax* sp. sequences, which were used as an outgroup. The final *MT-CYTB* dataset comprised 62 individual sequences, 45 *N. leucodon*, four *N. xanthodon*, eight *N. ehrenbergi*, and five *Spalax* sp. for the outgroup. They were subsequently aligned using ClustalW in MEGA Ver. X software [38].

Species identification—Estimates of evolutionary divergence over sequence pairs were performed between eight groups, i.e., five different cryptic species of the *N. leucodon* species complex, *N. xanthodon*, *N. ehrenbergi* and the outgroup (*Spalax* sp.), for each mtDNA gene separately in MEGA X, using the maximum composite likelihood model between groups with 10,000 bootstraps. Analyses were performed using the Kimura two-parameter model [39]. Rate variation between sites was modelled with a gamma distribution (shape parameter = 1). The two final data sets analysed comprised 62 nucleotide sequences, with a total of 492 and 425 positions for *16srRNA* and *MT-CYTB*, respectively. The codon positions included were 1st + 2nd + 3rd + Noncoding. All ambiguous positions were removed for each sequence pair (pairwise deletion option).

We applied two different phylogenetic analysis methods to confirm the strength of the tree topology: maximum likelihood (ML) in PhyML [40] and Bayesian analysis in MrBayes [41]. Phylogenetic trees were drawn using FigTree Ver. 1.3.1 (http://tree.bio.ed.ac.uk/software/figtree/) (accessed on 6 February 2024) and MEGA X. Prior to ML phylogenetic analysis, a best-fit substitution model in aligned sequences with the Akaike Information Criterion corrected for small sample sizes (AICc) [42] was established using jModelTest v.2.1.4. [40,43]. BI analyses originated with random starting trees and were run for 1 × 10^6^ generations, sampling every 100th generation, with the burn-in value set to 500. Combined trees from the various runs produced a 50% majority-rule consensus tree with the Bayesian posterior probability values of the relevant branches.

## 3. Results

### 3.1. Cryptic Species Identification

#### 3.1.1. *16srRNA* Gene Polymorphism

The obtained sequences are deposited in the GenBank database under the accession numbers PP349436–PP349461 (Table S1). The total data set contains 22 sequences that are free of stop codons, insertions, or deletions. In the 492 bp data set (485 excluding sites with gaps/missing data), there were 105 variable (polymorphic) sites, with 88 being parsimony informative, and a total of 135 mutations. A summary of the findings follows: total number of haplotypes, *h* = 34; nucleotide diversity, Pi: 0.05207; haplotype diversity, Hd = 0.9513 (variance of Hd = 0.0002, SD = 0.015).

The evolutionary divergence values calculated in MEGA X (Table 1) between the genera *Spalax*/*Nannospalax* ranged from 0.0939 to 0.1429; among three *Nannospalax* superspecies, *N. leucodon* was closer to *N. xanthodon* (0.063 to 0.0854) and *N. xanthodon* was closer to *N. ehrenbergi* (0.1024 to 0.1172). Among the five *N. leucodon* cryptic species distributed in Serbia, the lowest divergence (0.0140) was that between *N. l. syrmiensis* and *N. l. serbicus* and the highest (0.0537) was that between *N. l. hungaricus* and *N. l. montanosyrmiensis*. The lowest values of evolutionary divergence were found in *N. ehrenbergi* supersp.: that of *N. golani* and *N. galili* was 0.0052, and that of *N. judaei* and *N. carmeli* was 0.0136.

**Figure 2:**
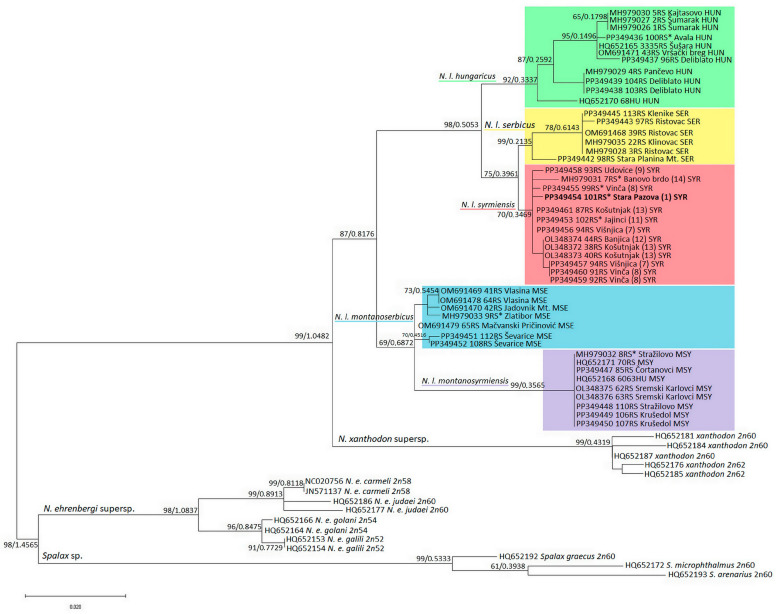
ML phylogenetic tree constructed using *16SrRNA* segment nucleotide polymorphism analysis. Similar topologies were inferred using the ML and BI methods; therefore, the support values were placed at the tree nodes. The sequences with asterisks are old, archived karyotyped samples (Collection Ivo Savić, IBISS). The numbers in brackets for *N. l. syrmiensis* correspond to the locality numbers in Figure 1c.

The topologies of the two trees, the ML phylogenetic tree generated using the Maximum Likelihood method with the TRN + I + G model suggested by jModelTest and the BI tree, are identical; therefore, only the ML tree with nodes supporting values from both methods is shown (Figure 2). The tree is rooted in the genus *Spalax* and shows a strongly supported division into two groups, the first being *N. ehrenbergi*, which is more closely related to *Spalax* sp., and the second being the sister position of *N. xanthodon* and *N. leucodon*, which are grouped into one clade. Each of the five *N. leucodon* cryptic species forms a distinct clade with high significance. There are two main clades, the first containing the phylogenetically older *N. l. montanosyrmiensis* and *N. l. montanoserbus* in the basal position, and the second containing *N. l. syrmiensis*, *N. l. serbicus,* and the youngest branch, *N. l. hungaricus*. All 18 sequences of newly collected individuals are grouped together with old, archived tooth-sample sequences of the same cryptic species. The tooth sample of a specimen from Avala Mt. (10 in Figure 1c), identified as *N. l. hungaricus* in the past, is clustered with other recent *N. l. hungaricus* specimens. Four tooth samples (1, 8, 11, and 14 in Figure 1c) from specimens previously determined by karyotyping to be *N. l. syrmiensis*, including the sample from the *locus typicus* Stara Pazova (1 in Figure 1c), are grouped with recently collected animals from the Belgrade (7, 8, 12, and 13 in Figure 1c) and Smederevo (9 in Figure 1c) regions. Our new samples (15 and 16 in Figure 1c) from the Mačva district (Ševarice near Bogatić) are grouped with other *N. l. montanoserbicus* samples from recently collected animals, but also with a tooth sample from a specimen captured in 1965 from Zlatibor Mt. The tooth sample of an individual from Stražilovo (on the slopes of Fruška Gora), *N. l. montanosyrmiensis*, is grouped with all other fresh samples from nearby localities, as well as from Kelebia. This cryptic species is the most homogeneous clade of *N. leucodon* analysed here.

#### 3.1.2. *MT-CYTB* Gene Polymorphism

The obtained sequences were deposited in the GenBank database under the accession numbers PP378178–PP378201 (Table S1). The total dataset comprises 20 sequences that are free of stop codons, insertions, or deletions. In the 425 bp dataset (424 excluding sites with gaps/missing data), there were 138 variable (polymorphic) sites, of which 126 were parsimony informative, and a total of 180 mutations were found. A summary of the findings follows: total number of haplotypes, *h* = 37; nucleotide diversity, Pi: 0.08913; haplotype diversity, Hd = 0.975 (variance of Hd = 0.00007, Sd = 0.008).

The values of evolutionary divergence generated from *MT-CYTB* polymorphism analysis (Table 2) are about twice as high as those derived from the *16SrRNA* gene, but the same relationships were found between the groups. Values of divergence among the genera *Spalax*/*Nannospalax* ranged from 0.2042 to 0.2380; *N. leucodon* was again found to be closely related to *N. xanthodon* (0.1020 to 0.1269) and *N. xanthodon* to *N. ehrenbergi* (0.1326 to 0.1416). The lowest divergence value, 0.0314, was found between *N. l. syrmiensis* and *N. l. serbicus*, but the highest value, 0.1073, was between *N. l. syrmiensis* and *N. l. montanosyrmiensis*. The lowest values of evolutionary divergence were again found in the *N. ehrenbergi* supersp.: *N. golani* and *N. galili*, 0.0183 and *N. judaei* and *N. carmeli,* 0.0195.

**Figure 3:**
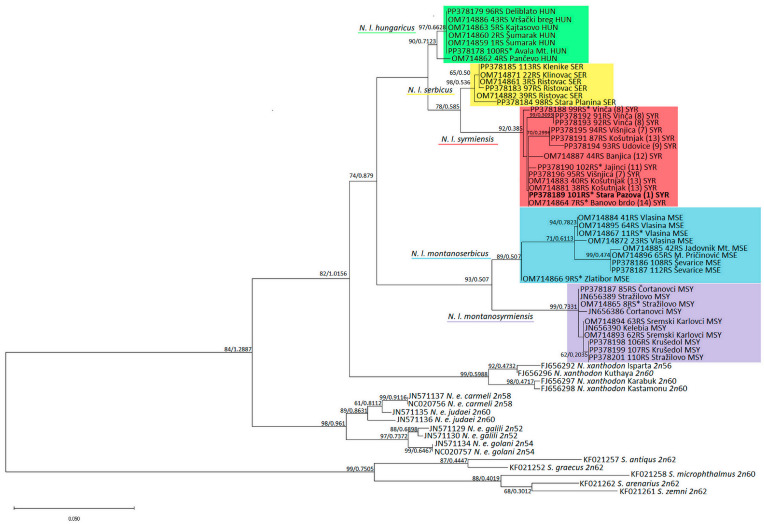
ML phylogenetic tree constructed using nucleotide polymorphism analysis of *MT-CYTB* gene sequences. Similar topologies were inferred using ML and BI methods; therefore, the support values were placed at the tree nodes. Sequences with asterisks are old, archived karyotyped samples (Collection Ivo Savić, IBISS). The numbers in brackets for *N. l. syrmiensis* correspond to the locality numbers in Figure 1c.

Similarly, the topologies of the ML phylogenetic tree generated using the HKY + I + G model from jModelTest and the BI tree are identical, so the support values of both methods were placed at the tree nodes in the ML tree (Figure 3). The topology of the *MT-CYTB* tree corresponds to *16SrRNA* rooted in *Spalax* sp. All five *N. leucodon* cryptic species, are divided into five clusters with high significance. The phylogenetically older *N. l. montanosyrmiensis* and *N. l. montanoserbicus* are in the basal position of the *N. leucodon* group. *N. l. syrmiensis*, *N. l. serbicus*, and *N. l. hungaricus* are closer to each other. An old, archived tooth sample from a specimen from Avala Mt. (10 in Figure 1c) is clustered with *N. l. hungaricus*. The sequences from four tooth samples (1, 8, 11, and 14 in Figure 1c) from specimens identified as *N. l. syrmiensis* by karyotyping in the past, including the sample from the *locus typicus* Stara Pazova (1 in Figure 1c), are clustered with samples from recently captured animals from the Belgrade (7, 8, 12, and 13 in Figure 1c) and Smederevo (9 in Figure 1c) regions. Fresh samples from localities near Bogatić (15 and 16 in Figure 1c) are clustered with the tooth sample of an animal from Vlasina and other newly collected *N. l. montanoserbicus* samples. The old, archived sample from Stražilovo (Fruška Gora Mt.), which was determined by karyotyping to be *N. l. montanosyrmiensis*, is clustered with newer samples from nearby localities and from Kelebia.

### 3.2. Presence and Distribution of N. l. syrmiensis

As a result of the five-year survey of localities attributed to *N. l. syrmiensis* (Stara Pazova (*locus typicus*), Višnjica, Banovo Brdo, Košutnjak, Avala, Jajinci, Smederevo, Vinča, and Bogatić [11]) and nearby areas, we report habitat loss, i.e., a greatly reduced distribution range of *N. l. syrmiensis* (Figure 1c). *Nannospalax l. syrmiensis* is no longer present at the Stara Pazova locality (*locus typicus*) (locality 1 in Figure 1c) or in the wide surroundings in the district of Srem (Vojvodina province) (localities 2–6 in Figure 1c) due to intensive anthropogenic activities. After extensive field work in cooperation with citizens, only moles were found in the entire area. On the slopes of Mount Avala (locality 10 in Figure 1c), the site of Beli Potok, where *N. l. syrmiensis* and *N. l. hungaricus* lived sympatrically in the same area (more precisely, in the same meadow), has now been destroyed by the massive construction of a highway.

We were able to confirm the occurrence of *N. l. syrmiensis* only in the Belgrade region, i.e., in Višnjica, Vinča, Banjica, and Košutnjak (7, 8, 12, and 13 in Figure 1c) and at the easternmost limit of its geographical range—Udovice, near Smederevo (locality 9 in Figure 1c). However, the westernmost part of its range in the Mačva district (Mačvanski Pričinović and Ševarice, both near Bogatić; 15 and 16 in Figure 1c) is now inhabited by another cryptic species, *N. l. montanoserbicus*.

## 4. Discussion

Here, we conducted five years of systematic field work, covering the entire known distribution range of *N. l. syrmiensis* [11,19]. For species identification, we compared the nucleotide sequences of two mitochondrial gene segments (*16SrRNA* and *MT-CYTB*) between newly collected animals and 46–61-year-old archived tooth samples that had been previously identified by karyotyping. For the species identity of *N. l. syrmiensis*, we used the tooth sample from the *locus typicus*—Stara Pazova (1 in Figure 1c)—together with three tooth samples from the Belgrade region—Vinča, Jajinci, and Banovo Brdo (8, 11, and 14 in Figure 1c).

Both genes yielded almost identical topologies using two different molecular phylogenetic methods (BI and ML), a result that is consistent with formerly published phylogenies [15,16,20,21]. The estimates of evolutionary divergence confirmed the relationships in the phylogenetic trees. As expected, the divergences inferred from the *MT-CYTB* dataset were about twice as high as those from the *16SrRNA* dataset because these two genes are functionally different and have different mutation rates. All sequences of *N. leucodon* derived from recently sampled animals were grouped into five clusters, corresponding to five cryptic species distributed in Serbia. These five clusters were divided into two main groups: an evolutionarily older branch with *N. l. montanosyrmiensis* and *N. l. montanoserbicus* in the basal position, and a younger branch with *N. l. serbicus*, *N. l. syrmiensis*, and *N. l. hungaricus*. This topology is consistent with the most recent *N. leucodon* phylogeny, with the exception of the placement of *N. l. syrmiensis*, which was marked as a missing taxon and “*incertae sedis*” [16]. Based on our results from both datasets, *N. l. serbicus* is the cryptic species closest to *N. l. syrmiensis*, but they are clearly separated in the phylogenetic trees, with high support. Moreover, the estimated evolutionary divergences between them exceed those between the only four species recognized in the literature, *Spalax* (=*Nannospalax*) *galili*, *S. golani*, *S. carmeli*, and *S. judaei*, which belong to *N. ehrenbergi* supersp. [9,12,44].

Finally, using a phylogenetic tree derived from both mtDNA markers, we have provided evidence that *N. l. syrmiensis* is not extinct in Serbia. Old, archived tooth samples (1, 8, 11, and 14 in Figure 1c) clustered with newly collected material, as expected according to karyotyping and known distribution areas [11], with the exception of samples from Mačva district (localities near Bogatić; 15 and 16 in Figure 1c). Mačva was designated as the westernmost part of the distribution range of *N. l. syrmiensis*, but our results confirmed the occurrence of *N. l. montanoserbicus* in this region. Thus, the distribution range of *N. l. montanoserbicus* is larger than previously defined and includes not only localities > 1000 m altitude [45], but also those at lower altitudes (e.g., ~60 m).

Historical overview of *N. l. syrmiensis*—Historically, the name “*syrmiensis*” (lat. *Syrmium* = Srem, a district in the province of Vojvodina between the rivers Sava in the south and Danube in the north) emerged as a result of an early taxonomy based solely on morphological data, when Méhely [46,47] named *Spalax monticola syrmiensis* as a separate species based on the examination of seven skulls from three localities in the district of Srem (Vojvodina, Serbia): Stara Pazova—*locus typicus*, Ruma and Sremska Mitrovica, but also a skull from the locality of Lelle (Somogy County, Hungary) in the western part of the Carpathian Basin. Only later, comprehensive karyotyping of BMR specimens [18,19,23] established that the Balkan region was inhabited by different CFs (with 2*n* = 46–56). Eight different species were proposed, including *Spalax leucodon syrmiensis* (Méhely 1909), as the monogeneric nomenclature was still in use. After summarizing the differences in chromosome number and morphology, evidence of reproductive isolation, and ecogeographic features, Savić and Soldatović [11] reported that the genus *Nannospalax* comprises 14 different species with subspecies. They described the species *Nannospalax syrmiensis* (Méhely 1909) with the karyotypic formula 2*n* = 54, *NF* = 90 as a group of seven populations endemic to Serbia, inhabiting the plains of the Srem district and the right bank of the Sava and Danube rivers from the confluence of the Drina, with the Sava in the west and the Great Morava with the Danube in the east [11]. Later, the nomenclature *Nannospalax leucodon syrmiensis* was accepted and widely used in the literature [6,8,10,14,15,16,21,26].

In contrast to BMRs from Srem District (Vojvodina, Serbia), which were confirmed as *N. l. syrmiensis* by karyotyping [11], the existence of *N. l. syrmiensis* in Somogy County (Hungary) has never been proven. Although it was emphasized that the BMRs were not sampled during the extensive cytogenetic mapping in the 1970s and that no karyological information on the populations from the Carpathian Basin is available [10], *N. l. syrmiensis* was declared extinct in Hungary and possibly extinct in Serbia [15]. Such conclusions may have a negative impact on the conservation of biodiversity, especially the genetic diversity of wildlife.

Chromosomal speciation and cryptic species in the genus *Nannospalax*—Chromosomal rearrangements are common in rodents, especially in the two most species-rich families, the Cricetidae and Muridae [48], and in the Ctenomyidae [49]. The theoretical basis for speciation mechanism through chromosomal rearrangements is described in detail. These rearrangements have the potential to drive post-zygotic isolation and gradually reduce gene flow, which makes them an important driver of the origination of cryptic species [50,51,52,53]. Chromosomal speciation within the lesser BMRs from the genus *Nannospalax* represents one of the few examples in nature [53,54,55,56]. It is characterized by extensive chromosomal rearrangements, i.e., karyotype variability. Pre- and post-copulation reproductive isolation between seven cryptic *N. leucodon* species, including *N. l. syrmiensis*, was demonstrated by comprehensive experimental crossbreeding and additionally confirmed by artificial insemination performed in similar combinations. Embryos developed only when individuals of the same cryptic species were united [8,11,19]. Reproductive isolation between the cryptic species of *N. leucodon*, which was caused by rapid chromosomal evolution, led to the deep genetic divergence reported in [14,15,16,21] and in this study. The unclear number of species and uncertain taxonomy have resulted in their inappropriate conservation status. Although many of them are seriously endangered, their status in the IUCN Red List of Threatened Species remains “Data Deficient” (DD) [13]. For the conservation of wildlife genetic diversity, it is important to acknowledge each cryptic species as a group of subpopulations that are genetically and ecologically differentiated [57].

*Nannospalax l. syrmiensis*—habitat loss and conservation perspectives—Besides cryptic species identification, after a detailed survey of localities formerly attributed to *N. l. syrmiensis* [11] and nearby areas, we found that the distribution range of *N. l. syrmiensis* has been highly reduced (Figure 1c). *N. l. syrmiensis* does not occur at the locality of Stara Pazova (*locus typicus*) (1 in Figure 1c) or in the wide surroundings in the district of Srem (Vojvodina province) (2–6 in Figure 1c). After detailed field work in cooperation with citizens, we found only moles in the whole area, including in steppe fragments near the villages of Donji Petrovac and Krnješevci (2 and 3 in Figure 1c). The species *N. l. syrmiensis* is confirmed only in the Belgrade region (7, 8, 12, and 13 in Figure 1c) and Udovice (9 in Figure 1c) near Smederevo, i.e., at the easternmost border of its geographical area—entry of the Great Morava River into the Danube. In the westernmost part of the area, at the entry of the Drina into the Sava in the Mačva district (15 and 16 in Figure 1c), we recorded only the cryptic species *N. l. montanoserbicus*.

Of all five cryptic species in Serbia, *N. l. syrmiensis* has the smallest distribution area. Such conditions, in combination with habitat fragmentation, lead to a decline in the number of individuals in populations, to their mutual isolation and frequently to complete extinction. The main risk factors for this cryptic species endemic to Serbia, according to the IUCN—CMP Unified Classification of Direct Threats [58], are as follows: urbanization and infrastructure construction, formation of agroecosystems and transition to intensive agricultural production, road construction, burning of vegetation, systematic transition from small-scale extensive production to large-scale intensive production systems, and habitat pollution, as previously published in [21]. As BMRs are herbivores, they often approach human gardens, farms, and parks, where they are treated as pests. Excessive killing and the use of chemical agents significantly increase the negative pressure on this cryptic species [11,21]. It is therefore necessary to investigate its population structure, to conduct a risk assessment, and to implement the conservation actions proposed by the IUCN—CMP Unified Classification of Conservation Actions Needed [59]. In particular, the following actions are needed: application of protection measures arising from their endemism to preserve their gene pool, informing the local residents about the level of protection of this mammal species and ways to protect their households without killing them, increasing the competence and efficiency of existing protection and monitoring services, creating and funding scientific teams that would increase the amount of research being conducted on insufficiently studied taxa and areas, and creating conditions to increase the diversity of mammal fauna in areas with intensive agricultural production [21].

## 5. Conclusions

For the conservation of biodiversity, including the genetic diversity of wildlife, declaring a particular cryptic species as probably extinct may have negative consequences. Through five years of field work and molecular genetic studies, we were able to provide evidence that *N. l. syrmiensis* is not extinct, as was reported in the literature. However, its habitat has been fragmented and reduced in size, primarily due to anthropogenic impact. Consequently, this cryptic species is on the retreat and certain conservation actions are required. For the preservation of wildlife genetic diversity, it is important to acknowledge each cryptic species. Many of these species are seriously endangered, while their status is still classified as Data Deficient (DD) in the IUCN Red List of Threatened Species. It is therefore crucial to clarify their taxonomy in order to assign to them the correct conservation status.

## Figures and Tables

**Table 1:** Estimates of evolutionary divergence over sequence pairs between groups using *16SrRNA* gene polymorphism dataset. Standard error estimates are shown above the diagonal.

	*HUN*	*SER*	*MSY*	*MSE*	*SYR*	*N. xan.*	*N. e. go.*	*N. e. ga.*	*N. e. ju.*	*N. e. ca.*	*Spalax*
*N. l. hungaricus*		0.0074	0.0110	0.0096	0.0074	0.0146	0.0169	0.0179	0.0181	0.0180	0.0191
*N. l. serbicus*	**0.0304**		0.0111	0.0095	0.0048	0.0144	0.0170	0.0182	0.0180	0.0176	0.0193
*N. l. montanosyrmiensis*	0.0537	0.0522		0.0073	0.0104	0.0136	0.0169	0.0170	0.0167	0.0170	0.0180
*N. l. montanoserbicus*	0.0444	0.0422	0.0254		0.0086	0.0120	0.0151	0.0163	0.0164	0.0160	0.0173
*N. l. syrmiensis*	**0.0301**	**0.0140**	0.0459	0.0353		0.0141	0.0168	0.0180	0.0177	0.0173	0.0178
*N. xanthodon*	0.0854	0.0827	0.0735	0.0630	0.0788		0.0167	0.0168	0.0179	0.0169	0.0201
*N. e. golani*	0.1021	0.1029	0.0996	0.0862	0.0992	0.1024		0.0031	0.0086	0.0080	0.0154
*N. e. galili*	0.1100	0.1124	0.1006	0.0953	0.1086	0.1027	0.0052		0.0088	0.0087	0.0163
*N. e. judaei*	0.1186	0.1175	0.1023	0.1035	0.1135	0.1172	0.0367	0.0379		0.0043	0.0148
*N. e. carmeli*	0.1139	0.1090	0.1010	0.0953	0.1051	0.1046	0.0288	0.0322	0.0136		0.0149
*Spalax* sp.	0.1325	0.1324	0.1197	0.1157	0.1197	0.1429	0.0975	0.1049	0.0970	0.0939	

bold—values between *N. l. syrmiensis* and two closest cryptic species *N. l. hungaricus* and *N. l. serbicus*; red—the lowest values found between four species of *N. ehrenbergi* supersp.

**Table 2:** Estimates of evolutionary divergence over sequence pairs between groups using the *MT-CYTB* gene polymorphism dataset. Standard error estimates are shown above the diagonal.

	*SYR*	*MSY*	*MSE*	*SER*	*HUN*	*N. xan.*	*N. e. ju.*	*N. e. ca.*	*N. e. ga.*	*N. e. go.*	*Spalax*
*N. l. syrmiensis*		0.0188	0.0179	0.0091	0.0109	0.0203	0.0234	0.0239	0.0237	0.0240	0.0301
*N. l. montanosyrmiensis*	0.1073		0.0140	0.0179	0.0159	0.0206	0.0253	0.0272	0.0258	0.0262	0.0291
*N. l. montanoserbicus*	0.1039	0.0724		0.0168	0.0155	0.0178	0.0204	0.0214	0.0207	0.0210	0.0266
*N. l. serbicus*	**0.0383**	0.0991	0.0952		0.0084	0.0184	0.0207	0.0213	0.0216	0.0213	0.0287
*N. l. hungaricus*	**0.0482**	0.0830	0.0835	**0.0314**		0.0178	0.0186	0.0193	0.0204	0.0199	0.0261
*N. xanthodon*	0.1241	0.1269	0.1106	0.1087	0.1020		0.0208	0.0211	0.0224	0.0215	0.0256
*N. e. judaei*	0.1459	0.1624	0.1258	0.1255	0.1075	0.1333		0.0062	0.0114	0.0118	0.0260
*N. e. carmeli*	0.1471	0.1736	0.1309	0.1267	0.1087	0.1311	0.0195		0.0127	0.0131	0.0267
*N. e. galili*	0.1461	0.1643	0.1264	0.1296	0.1176	0.1416	0.0515	0.0555		0.0065	0.0267
*N. e. golani*	0.1477	0.1663	0.1274	0.1248	0.1134	0.1326	0.0529	0.0570	0.0183		0.0261
*Spalax* sp.	0.2380	0.2287	0.2119	0.2265	0.2015	0.2020	0.2042	0.2074	0.2083	0.2028	

bold—values between *N. l. syrmiensis* and two closest cryptic species; red—the lowest values of evolutionary divergence.

## Data Availability

DNA nucleotide sequences were deposited in the NCBI GenBank, and can be assessed at https://www.ncbi.nlm.nih.gov/genbank, accessed on 26 February 2024.

## Supplementary Materials

The following supporting information can be downloaded at: https://www.mdpi.com/article/10.3390/ani14050774/s1, Table S1: A list of 22 new *N. leucodon* and samples imported from GenBank, with IDs (GenBank accession numbers) for mtDNA gene sequences. 2*n*—diploid chromosomal numbers; *NF*—fundamental number of chromosomal arms. ^is^—Collection Ivo Savic, IBISS; ISO—ISO country code.
