# Supplementary material for: A Cryptic Subterranean Mammal Species, the Lesser Blind Mole Rat (Nannospalax leucodon syrmiensis)—Retreated but Not Extinct
Source: Animals (Basel). 2024 Feb 29;14(5):774. doi: 10.3390/ani14050774 (PMC10931004; doi:10.3390/ani14050774)
Supplement: Supplementary file 1 [file animals-14-00774-s001.zip › animals-2895218-supplementary.pdf]

Table S1. A list of 22 new *N. leucodon* and samples imported from GenBank, with IDs (GenBank accession numbers) for mtDNA gene sequences. 2*n*—diploid chromosomal numbers; *NF*—fundamental number of chromosomal arms. [is] - Collection Ivo Savic, IBISS; ISO – ISO country code.

16S rRNA ID	*MT-CYTB* ID	Sample No	Sampling locality, ISO	CF/species	2*n*/*NF*	Reference *16SrRNA*/*MT-CYTB*
PP349447	PP378197	85RS	Čortanovci, RS	*montanosyrmiensis*	54/86	This study
PP349461	PP378191	87RS	Košutnjak, RS	*syrmiensis*	54/90	This study
PP349460	PP378192	91RS	Vinča, RS	*syrmiensis*	54/90	This study
PP349459	PP378193	92RS	Vinča, RS	*syrmiensis*	54/90	This study
PP349458	PP378194	93RS	Udovice, RS	*syrmiensis*	54/90	This study
PP349457	PP378195	94RS	Višnjica, RS	*syrmiensis*	54/90	This study
PP349456	PP378196	95RS	Višnjica, RS	*syrmiensis*	54/90	This study
PP349437	PP378179	96RS	Deliblato, RS	*hungaricus*	48/84	This study
PP349443	PP378183	97RS	Ristovac, RS	*serbicus*	54/98	This study
PP349442	PP378184	98RS	Stara Planina, RS	*serbicus*	54/98	This study
PP349455	PP378188	99RS [is]	Vinča, RS	*syrmiensis*	54/90	This study
PP349436	PP378178	100RS [is]	Avala, RS	*hungaricus*	48/84	This study
PP349454	PP378189	101RS [is]	Stara Pazova, RS	*syrmiensis*	54/90	This study
PP349453	PP378190	102RS [is]	Jajinci, RS	*syrmiensis*	54/90	This study
PP349438	/	103RS	Deliblato, RS	*hungaricus*	48/84	This study
PP349439	/	104RS	Deliblato, RS	*hungaricus*	48/84	This study
PP349449	PP378198	106RS	Krušedol, RS	*montanosyrmiensis*	54/86	This study
PP349450	PP378199	107RS	Krušedol, RS	*montanosyrmiensis*	54/86	This study
PP349452	PP378186	108RS	Ševarice, RS	*montanoserbicus*	56/82	This study
PP349448	PP378201	110RS	Stražilovo, RS	*montanosyrmiensis*	54/86	This study

PP349451	PP378187	112RS	Ševarice, RS	*montanoserbicus*	56/82	This study
PP349445	PP378185	113RS	Klenike, RS	*serbicus*	54/98	This study
MH979026	OM714859	1RS	Šumarak , RS	*hungaricus*	48/84	Bugarski-Stanojević et al. 2020/2022
MH979027	OM714860	2RS	Šumarak, RS	*hungaricus*	48/84	Bugarski-Stanojević et al. 2020/2022
MH979028	OM714861	3RS	Ristovac, RS	*serbicus*	54/98	Bugarski-Stanojević et al. 2020/2022
MH979029	OM714862	4RS	Pančevo, RS	*hungaricus*	48/84	Bugarski-Stanojević et al. 2020/2022
MH979030	OM714863	5RS	Kajtasovo, RS	*hungaricus*	48/84	Bugarski-Stanojević et al. 2020/2022
MH979031	OM714864	7RS[is]	Beograd (Banovo brdo), RS	*syrmiensis*	54/90	Bugarski-Stanojević et al. 2020/2022
MH979032	OM714865	8RS[is]	Stražilovo, RS	*montanosyrmiensis*	54/86	Bugarski-Stanojević et al. 2020/2022
MH979033	OM714866	9RS[is]	Zlatibor Mt, RS	*montanoserbicus*	56/82	Bugarski-Stanojević et al. 2020/2022
/	OM714867	11RS[is]	Vlasina, RS	*montanoserbicus*	56/82	Bugarski-Stanojević et al. 2022
MH979035	OM714871	22RS	Klinovac, RS	*serbicus*	54/98	Bugarski-Stanojević et al. 2020/2022
/	OM714872	23RS	Vlasina, RS	*montanoserbicus*	56/82	Bugarski-Stanojević et al. 2022
OL348372	OM714881	38SL	Beograd (Košutnjak), RS	*syrmiensis*	54/90	Bugarski-Stanojević et al. 2022
OM691468	OM714882	39RS	Ristovac, RS	*serbicus*	54/98	Bugarski-Stanojević et al. 2022

OL348373	OM714883	40RS	Beograd (Košutnjak), RS	*syrmiensis*	54/90	Bugarski-Stanojević et al. 2022
OM691469	OM714884	41RS	Vlasina, RS	*montanoserbicus*	56/82	Bugarski-Stanojević et al. 2022
OM691470	OM714OM885	42RS	Jadovnik Mt. (Prijepolje), RS	*montanoserbicus*	56/82	Bugarski-Stanojević et al. 2022.
OM691471	OM714886	43RS	Vršački breg, RS	*hungaricus*	48/84	Bugarski-Stanojević et al. 2022
OL348374	OM714887	44RS	Beograd (Banjica), RS	*syrmiensis*	54/90	Bugarski-Stanojević et al. 2022
OL348375	OM714893	62RS	Sremski Karlovci (Stražilovo), RS	*montanosyrmiensis*	54/86	Bugarski-Stanojević et al. 2022
OL348376	OM714894	63RS	Sremski Karlovci (Stražilovo), RS	*montanosyrmiensis*	54/86	Bugarski-Stanojević et al. 2022.
OM691478	OM714895	64RS	Vlasina, RS	*montanoserbicus*	56/82	Bugarski-Stanojević et al. 2022
OM691479	OM714896	65RS	Mačvanski Pričinović, RS	*montanoserbicus*	56/82	Bugarski-Stanojević et al. 2022
HQ652170	/		Mezőtúr, HU	*hungaricus*	48/84	Hadid et al. 2012
HQ652165	/		Šušara, RS	*hungaricus*	48/84	Hadid et al. 2012
HQ652171	/		Subotička Peščara, RS	*montanosyrmiensis*	54/86	Hadid et al. 2012
HQ652168	/		"isolate 6063"	*montanosyrmiensis*	54/86	Hadid 2010
HQ652184	/		Karaman, TU	*N. xanthodon*	60	Hadid et al. 2012
HQ652185	/		Konya, TU	*N. xanthodon*	62	Hadid et al. 2012
HQ652187	/		Malatya, TU	*N. x. vasvarii*	60	Hadid et al. 2012
HQ652176	/		Ankara, TU	*N. xanthodon*	62	Hadid et al. 2012
HQ652181	/		Denizili, TU	*N. xanthodon*	60	Hadid et al. 2012
HQ652154	/		Alma, IL	*N. e. galili*	52	Hadid et al. 2012

HQ652153	/	Alma, IL	*N. e. galili*	52	Hadid et al. 2012
HQ652164	/	Mt. Hermon, IL	*N. e. golani*	54	Hadid et al. 2012
HQ652166	/	El Al, IL	*N. e. golani*	54	Hadid et al. 2012
HQ652177	/	Anza, IL	*N. e. judaei*	60	Hadid et al. 2012
HQ652186	/	Lahav, IL	*N. e. judaei*	60	Hadid et al. 2012
JN571137	/	Muhraka, IL	*N. e. carmeli*	58	Hadid et al. 2012
NC020756	/	Mt Carmel, IL	*N. e. carmeli*	58	Hadid et al. 2012
HQ652192	/	Iaşi, RO	*Spalax graecus*	62	Hadid 2010
HQ652193	/	Kherson, UA	*S. arenarius*	62	Hadid 2010
HQ652172	/	Novomoszkovsz, UA	*S. microphthalmus*	60	Hadid 2010
/	JN656390	Kelebia, RS	*montanosyrmiensis*	54/86	Nemeth et al. 2013
/	JN656389	Stražilovo, RS	*montanosyrmiensis*	54/86	Nemeth et al. 2013
/	JN656386	Čortanovci, RS	*montanosyrmiensis*	54/86	Nemeth et al. 2013
/	FJ656298	Kastamonu, TU	*N. xanthodon*	2n60	Kandemir et al. 2012
/	FJ656297	Karabük, TU	*N. xanthodon*	2n60	Kandemir et al. 2012
/	FJ656296	Kütahya, TU	*N. xanthodon*	2n60	Kandemir et al. 2012
/	FJ656292	Isparta, TU	*N. xanthodon*	2n56	Kandemir et al. 2012
/	NC020756	Carmel Mt, IL	*N. carmeli*	58	Hadid et al. 2012
/	JN571137	Carmel Mt, IL	*N. carmeli*	58	Hadid et al. 2012
/	JN571135	Judean Mt, IL	*N. judaei*	60	Hadid et al. 2012
/	JN571136	Judean Mt, IL	*N. judaei*	60	Hadid et al. 2012
/	JN571134	Golan Heights, IL	*N. golani*	54	Hadid et al. 2012
/	NC020757	Golan Heights, IL	*N. golani*	54	Hadid et al. 2012
/	JN571130	Galilee Mt, Il	*N. galili*	52	Hadid et al. 2012

/	JN571129	Galilee Mt, Il	*N. galili*	52	Hadid et al. 2012
/	KF021257	Sânduleşti, RO	*S. antiquus*	62	Nemeth et al. 2013
/	KF021252	David Valley, RO	*S. graecus*	62	Nemeth et al. 2013
/	KF021258	Novomoszkovsz, UK	*S. microphthalmus*	60	Nemeth et al. 2013
/	KF021262	Kherson, UK	*S. arenarius*	62	Nemeth et al. 2013
/	KF021261	Krivij Rig, UK	*S. zemni*	62	Nemeth et al. 2013
